# Understanding DNA Damage Response and DNA Repair in Multiple Myeloma

**DOI:** 10.3390/cancers15164155

**Published:** 2023-08-17

**Authors:** Cole Petrilla, Joshua Galloway, Ruchi Kudalkar, Aya Ismael, Francesca Cottini

**Affiliations:** Division of Hematology, Department of Internal Medicine, College of Medicine, The Ohio State University, Columbus, OH 43210, USA

**Keywords:** multiple myeloma, DNA damage response, DNA repair

## Abstract

**Simple Summary:**

Multiple myeloma (MM), a malignant plasma cell disorder, is characterized by abnormal DNA damage response (DDR). MM cells adapt over time via gene mutations or chromosomal aberrations, including changes to DDR and DNA repair genes. Increased DNA repair and avoidance of DNA-damaged induced cellular death promote tumor formation, progression, and resistance to treatments. Because of the wide array of DDR and DNA repair mechanisms, DDR is an elusive target. Currently, treatments such as proteasome inhibitors and alkylating agents are commonly used in patients with MM. These treatments affect DDR and DNA repair pathways in different ways. As more studies are conducted, targeting DDR mechanisms might emerge as new treatments, as described in this review.

**Abstract:**

Multiple myeloma (MM) is a plasma cell malignancy characterized by several genetic abnormalities, including chromosomal translocations, genomic deletions and gains, and point mutations. DNA damage response (DDR) and DNA repair mechanisms are altered in MM to allow for tumor development, progression, and resistance to therapies. Damaged DNA rarely induces an apoptotic response, given the presence of ataxia-telangiectasia mutated (*ATM*) loss-of-function or mutations, as well as deletions, mutations, or downregulation of tumor protein p53 (TP53) and tumor protein p73 (TP73). Moreover, DNA repair mechanisms are either hyperactive or defective to allow for rapid correction of the damage or permissive survival. Medications used to treat patients with MM can induce DNA damage, by either direct effects (mono-adducts induced by melphalan), or as a result of reactive oxygen species (ROS) production by proteasome inhibitors such as bortezomib. In this review, we will describe the mechanisms of DDR and DNA repair in normal tissues, the contribution of these pathways to MM disease progression and other phenotypes, and the potential therapeutic opportunities for patients with MM.

## 1. Introduction

Environmental stressors or biologic components such as reactive oxidative species (ROS) are known to induce mutations and genomic abnormalities [1]. Cancer cells, due to a high replication rate, oncogene activation, or ROS production, have a greater mutational burden than normal tissues [2]. As increasing genomic abnormalities emerge, cancer cells activate the DNA damage response (DDR) to either correct otherwise deteriorating DNA sequences (DNA repair pathways) or to undergo senescence or apoptosis [3]. Indeed, with the introduction of beneficial mutational abnormalities come unwanted gene mutations which hinder proliferation or survival. As DNA repair pathways are usually hyperactive in cancer cells, new inhibitors targeting these mechanisms are under development to stop or stall the progression of cancer by reducing DNA repair and hence increasing cell death [4].

**Figure 1:**
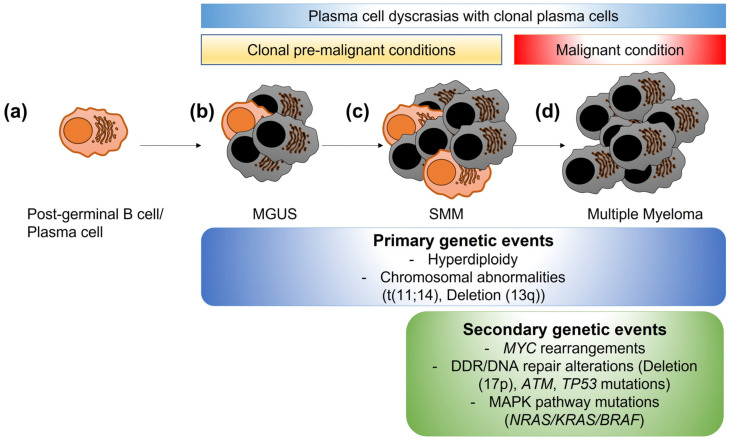
Clonal evolution of plasma cell dyscrasias. (**a**) A mature post-germinal B cell or plasma cell normally produces specific antibodies to aid in immune responses. (**b**) In some individuals, low levels of abnormal post-germinal B cells/plasma cells become clonally expanded (grey cells), producing a specific monoclonal-protein. This stage is called gammopathy of undetermined significance (MGUS) and is a premalignant condition. (**c**) Smoldering multiple myeloma (SMM) is the progression of MGUS, with higher numbers of abnormal clonal plasma cells. SMM is still a premalignant condition. (**d**) Multiple myeloma is the overt malignant condition, with higher percentages of abnormal clonal plasma cells leading to organ damage (anemia, kidney injury, bone lesions, or hypercalcemia). While some genetic changes are considered primary events also present in MGUS and SMM stages, secondary events, such as *MYC* rearrangements, alterations/mutations in DNA damage response or DNA repair genes, or mutations in the mitogen-activated protein kinase (MAPK) pathway genes are associated with progression to MM.

Multiple myeloma (MM) is a plasma cell malignancy originating in the bone marrow of afflicted individuals [5]. Over 35,000 individuals are diagnosed yearly with MM in the United States, with a median age of diagnosis of 68 years. MM is characterized by different clinical scenarios, including fatigue, lytic bone lesions, and kidney damage. MM is a disease with a multistep development pathway, beginning with healthy differentiated plasma cells. A preliminary step in MM is monoclonal gammopathy of undetermined significance (MGUS), where abnormal plasma cells start producing a specific monoclonal protein (Figure 1), followed by a phase called smoldering MM (SMM). Although not all MGUS develop into MM, all cases of MM arise from MGUS [6]. MM karyotypes are usually complex, with numerical (hyperdiplody or hypodiplody) and structural abnormalities, such as chromosomal translocations, gains, or deletions of genomic loci or wider areas [7]. Research into specific genomic abnormalities using fluorescence in situ hybridization (FISH) methods have identified a variety of genomic aberrations, such as chromosomal translocations (e.g., t(11;14), t(4;14), t(14;16)), chromosomal deletions (del(17p13) or del(13q) or chromosomal gains (1q21+). Some of them, including chromosomal translocation t(11;14) or deletion 13q, are considered primary genetic events, also occurring in MGUS; others are considered secondary genetic events associated with progression to overt MM, such as *MYC* (MYC proto-oncogene, bHLH transcription factor) rearrangements, abnormalities in the DDR/DNA damage pathway (e.g., ataxia-telangiectasia mutated-ATM deletion, tumor protein p53-TP53 mutations, del(17p)), or mutations in genes of the mitogen-activated protein kinase (MAPK) pathway (KRAS proto-oncogene, GTPase-*KRAS*, NRAS proto-oncogene, GTPase-*NRAS*, B-Raf proto-oncogene, serine/threonine kinase-*BRAF*) [8,9] (Figure 1). Despite several therapeutic options, MM remains incurable, with poor outcomes in patients with high-risk features [10]. Autologous stem cell transplants (ASCTs) using melphalan, an alkylating agent, are still widely used due to their progression-free survival benefits [11,12]. However, recent studies have shown that melphalan increases the general mutational burden in MM cells [13], reopening the debate about the balance between DNA damage-mediated apoptosis and the repair of damaged DNA.

In this review, we will discuss the role of DDR and DNA repair in MM and present therapeutic strategies to exploit this phenotype.

## 2. DDR and DNA Repair in Normal Tissues and in MM

### 2.1. DDR and DNA Repair in Normal Tissues

**Figure 2:**
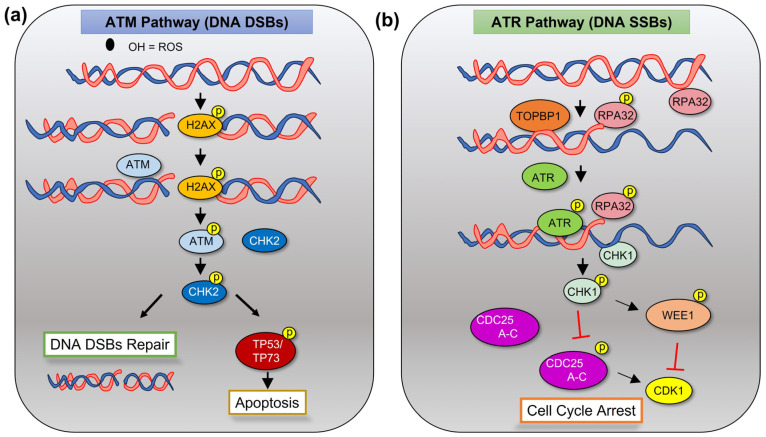
ATM- and ATR-mediated pathways in the presence of damaged DNA. (**a**) The ATM signaling pathway responds to DNA double-strand breaks (DSBs), which cause the inactive ATM to split into active ATM monomers to start a phosphorylation cascade. This event recruits other signaling proteins, such as CHK2 and TP53, to allow for DNA damage repair or apoptosis and senescence if the damage is too severe. (**b**) The ATR pathway is activated in times of replicative stress caused by DNA single-strand breaks (SSBs), lack of nucleotide bases, and stalls at the replication fork. The subsequent binding of replication protein A2 (RPA32) to the replication fork allows a phosphorylation cascade of ATR and CHK1 to happen, leading to cell cycle arrest by CDC25-mediated CDK1 activation to ensue DNA fidelity. p = phosphorylation.

DDR starts with recognition of damaged DNA by sensor proteins followed by activation of repair mechanisms, which differ based on the type of damage (Figure 2) [2]. ATM is the kinase involved in the repair of DNA double-strand breaks (DSBs), while ataxia telangiectasia and Rad3-related (ATR) is activated to relieve replication stress by DNA single-strand breaks (SSBs) [14]. ATM is activated by autophosphorylation into its monomeric form, where then it phosphorylates its downstream targets such as serine/threonine checkpoint kinase 2 (CHK2) [14]. Similarly, ATR, activated by topoisomerase binding protein 1 (TOPBP1), phosphorylates checkpoint kinase 1 (CHK1) in response to SSBs [15]. CHK1 and CHK2 then reduce cyclin-dependent kinase (CDK) activity, by either activating TP53 or inhibiting Ras family guanine nucleotide exchange factor cell division cycle 25 (CDC25A-C) phosphatases [16]. This leads to the attenuation or arrest of cell-cycle progression in order to stabilize replication forks, modulate proteins and enzymes involved with DNA repair, and either repair the damaged DNA or trigger terminal apoptosis or senescence if the damage is too extensive [17]. WEE1 G2 checkpoint kinase (WEE1) is another kinase involved in the protection of genome integrity. At the G2 phase of the cell cycle, WEE1 is phosphorylated by CHK1, becoming activated and preventing progression into the M phase until the damage is repaired [18].

**Figure 3:**
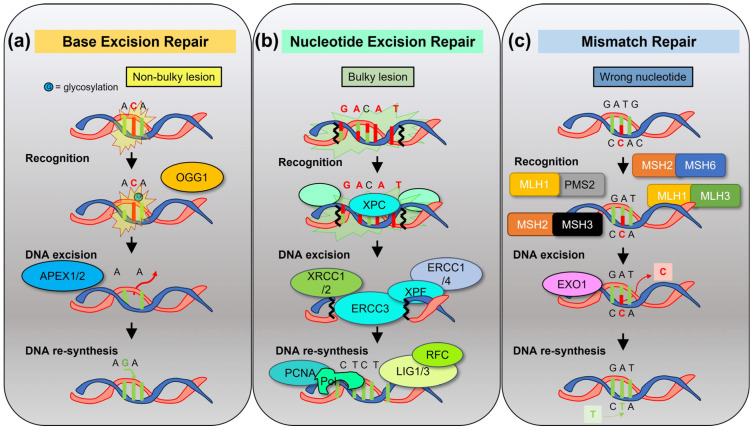
Excision repair pathways of DNA single-strand breaks. Three types of excision repair pathways exist to remove damaged single-strand DNA and re-synthesize a new DNA strand, using the undamaged complementary strand as a template. (**a**) Base excision repair (BER) is responsible for the removal of non-bulky DNA lesions due to oxidation, alkylation, or glycosylation. Damage is recognized by a DNA glycosylase, such as OGG1. The damaged portion is then resected by APEX1/2 enzymes to undergo new DNA synthesis. (**b**) Nucleotide excision repair (NER) occurs in the presence of bulky lesions to remove a string of nucleotides. After damaged DNA or helix distortion is recognized and verified by XPC, a transcription initiation and repair factor complex composed of ERCC1, ERCC3, ERCC4, and XRCC1 proteins is recruited, and new DNA is added by replication protein proliferating cell nuclear antigen (PCNA), replication factor C (RFC), DNA polymerases, and DNA ligase 1 or XRCC1–DNA ligase 3. (**c**) Mismatch repair is activated when an incorrect Watson–Crick base pairing (outside the typical A to T and G to C) occurs. Mutation recognition of errors is performed by heterodimers MSH2-MSH6 or MSH2-MSH3, followed by termination of mismatch-provoked excision by heterodimers MLH1-PMS2 or MLH1-MLH3, full excision by EXO1, and replacement with the correct base pairing.

**Figure 4:**
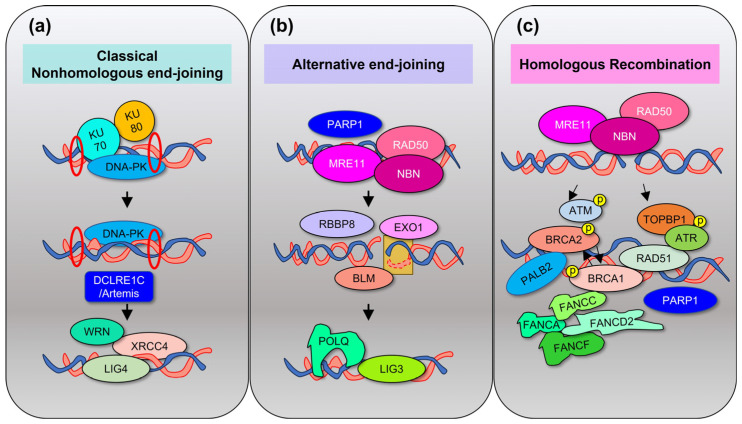
DNA repair pathways of DNA double-strand breaks. The classical nonhomologous end-joining (c-NHEJ) pathway, the alternative end-joining pathway (alt-EJ), and the homologous recombination (HR) pathway repair DNA double-strand breaks by ligating the broken DNA ends (c-NHEJ) using short homologous sequences after resection of the damaged DNA (alt-EJ) or reconstituting the original sequence (HR). (**a**). DNA-PK forms a complex with KU70/KU80 to activate DCLRE1C/Artemis and then promote excision/repair by XRCC4/LIG4. (**b**). PARP1 binds to the affected DNA and allows MRN complex to form. EXO1, BLM, and RBBP8 causes an extensive end resection which is repaired by POLQ and LIG3. (**c**). HR via ATM and ATR pathways activates BRCA1/2 and promotes recruitment of other proteins such as Fanconi proteins (FANCA, FANCC, FANCD2, and FANCF) and PARP1/2. p = phosphorylation.

Mammalian cells have several DNA repair pathways to prevent mutagenesis. The base excision repair (BER), nucleotide excision repair (NER), and mismatch repair (MMR) pathways act on nucleotide lesions on single-strand DNA (ssDNA) [19], using as a template the undamaged complementary strand (Figure 3). Conversely, the homologous recombination (HR) pathway, the classical nonhomologous end-joining (c-NHEJ) pathway, or the error-prone alternative end-joining (alt-EJ) pathways repair DNA DSBs with different rates of efficiency, potentially leading to chromosomal breakage and loss of genetic material [20] (Figure 4). In general, these pathways include enzymes involved in damage recognition, DNA excision, and DNA re-synthesis. BER eliminates base damages or non-bulky DNA lesions that occur spontaneously due to hydrolysis, oxidation, or other reactions after recognition by DNA glycosidases such as 8-oxoguanine DNA glycosylase (OGG1), followed by apurinic/apyrimidinic endodeoxyribonuclease 1 (APEX1)-mediated excision [21]. The NER pathway recognizes and removes bulky helix-disturbing DNA lesions induced by environmental stressors such as ultraviolet light and tobacco or by chemotherapies, such as the N-alkyl purine-mono adducts induced by melphalan [22]. The MMR pathway consists of four major proteins (mutL homolog 1-MLH1, mutS homolog 2-MSH2, mutS homolog 6-MSH6, and PMS1 homolog 2, mismatch repair system component-PMS2). The role of MMR is to correct mismatched nucleotide pairs and other insertion/deletion mutations to prevent recombination of divergent sequences and extension of harmful mutations into the genome and the genome of descending cells. Mutation sensing is carried out by different heterodimers: the MutSα heterodimer (MSH2-MSH6), for erroneously paired nucleotides, and the MutSβ heterodimer (MSH2-MSH3), for insertion/deletion mutations. These heterodimers then bind to other heterodimers such as MutLα (MLH1/PMS2) and MutLβ (MLH1/MLH3) to create ssDNA breaks which allow for the excision of the DNA mismatch by exonuclease 1 (EXO1) [23]. When the MutLα and MutSα heterodimers are separated into their monomeric states, the proteins are rapidly degraded, and expression loss of one monomer leads to the expression loss of the partner monomer.

Regarding the pathways involved in repair of DNA DSBs, NHEJ is active throughout the whole cell cycle, while HR acts preferentially in the S and G2 phases of the cell cycle, leading to differing accuracy in repair of the original DNA sequence [24] (Figure 4). In the NHEJ pathway, DNA DSBs are recognized by DNA-dependent protein kinase (DNA-PK), a serine/threonine protein kinase, which forms a complex with Ku proteins (Ku70/80). This activates DNA cleavage via DNA cross-link repair 1C (DCLRE1C/Artemis), phosphorylation of Werner syndrome helicase (WRN), and then activation of X-ray repair cross-complementing 4 (XRCC4) and DNA Ligase IV (LIG4) to ensure DNA repair. In cases of loss of Ku proteins, the alt-EJ pathway is activated, leading to extensive resection of the DNA ends to reveal sequence homology, followed by annealing and ligation via a complex composed of DNA polymerase theta (POLQ), Pol poly(ADP-ribose) polymerase 1 (PARP1), RB binding protein 8, endonuclease (RBBP8), Ligase 3 (LIG3), and the MRN protein complex (MRE11 homolog, double-strand break repair nuclease-MRE11, RAD50 double-strand break repair protein-RAD50, and nibrin-NBN) [20].

The HR pathway includes recruitment of MRN protein complex to DNA DSBs, activation of ATM/ATR, BRCA1 DNA repair-associated/BRCA2 DNA repair-associated/partner and localizer of BRCA2 (BRCA1/BRCA2/PALB2) with end resection to ssDNA, RAD51 recombinase (RAD51) recruitment, and DNA repair. Finally, the Fanconi anemia (FA)/BRCA pathway repairs interstrand cross-link (ICL) lesions, such as those created by melphalan, in cooperation with the NER and HR pathways [25]. The FA/BRCA pathway relies on FA-related proteins and BRCA1/2 pleiotropic functions to regulate checkpoint activation, DNA repair, and homologous recombination [26]. *BRCA1* and *BRCA2* mutations are known to predispose individuals to different types of hereditary cancers, such as breast and ovarian cancers [27,28,29]. Cancers that have lost the essential *BRCA* genes utilize WRN helicases to prevent stalled replication fork degradation [30] and rely on DNA repair facilitated by PARP1 and PARP2 [31]. Therefore, cancer cells that lack the *BRCA* genes or other HR genes are subjected to critically high levels of DSBs and are consequently more sensitive to PARP inhibition [31]. Programmed death-ligand 1 (PD-L1) has also been linked to HR DNA repair, promoting DNA end resection in cancer cells [32]. PD-L1 deficiency or downregulation, but not surface PD-L1 inhibition with monoclonal antibodies, was able to induce DNA damage accumulation and increase response to PARP inhibitors in *BRCA1* wild-type tumors, cisplatin, or radio-immunotherapy [32,33,34]. In turn, radiation or chemotherapeutic agents promote PD-L1 expression on the surface of cancer cells [35].

### 2.2. Preventing Apoptotic DNA Damage Response (DDR) in MM

Ongoing DNA damage and DNA DSBs are present in MM cells, as revealed by constitutive phosphorylation of H2A histone family member X (H2A.X) [36,37]. We previously showed that not only do DNA DSBs occur, but there is also constitutive activation of ATM/CHK2 kinases without eliciting an apoptotic response. This is largely due to the deletion and inactivation of yes-associated protein 1 (*YAP1*) leading to a dampened apoptotic response by ABL proto-oncogene 1, non-receptor tyrosine kinase (ABL1), and tumor protein p73 (TP73) [37]. Serine/threonine kinase 4 (STK4) inhibition was able to restore DNA-damage mediated apoptosis at baseline or upon treatment with doxorubicin; specific YAP1 activators also induce cell death in MM [38]. We then showed that replicative stress is present in MM with activation of ATR/CHK1 via oncogene addiction [39]. Specifically, *MYC* oncogene, which is dysregulated in MM due to gene rearrangements or overexpression [40,41,42], is able to induce activation of replicative stress markers with phosphorylation of ATR and replication protein A2 (RPA2/RPA32). This was proven in cell lines negative for *MYC* via re-introduction of the gene. Other oncogenes are activated in MM, with 40 percent of cases bearing gene mutations in *KRAS* or *NRAS* [7]. No data is available on RAS and replicative stress in MM, but ATR inhibition with VE-821 was able to induce cell death in cells overexpressing *MYC* and in cells with *RAS* mutations [39]. Moreover, a combination of melphalan, a drug used as a conditioning regimen for ASCT in myeloma, and ATR inhibitors was proven synergic in *in vitro* and *in vivo* models of MM [43]. Finally, *TP53* deletions or point mutations [44], as well as *TP73* promoter methylation [45], are common in MM, further reducing the ability to induce apoptosis or senescence of DNA-damaged cells.

### 2.3. DNA Repair Pathways in MM

Several DNA repair pathways are dysregulated in MM or are important for therapeutic responses. In general, upregulation of DNA repair mechanisms, especially by oncogenes such as *MYC*, are associated with aggressive myeloma and shorter response to ASCT [46].

Aberrancies in the repair of DNA SSBs have been reported in MM patients. APEX1 and/or APEX2 are the key BER proteins involved in the excision of non-bulky DNA lesions. The expression of APEX1 and APEX2 is upregulated in MM cell lines and in a subset of MM patient samples compared with normal plasma cells. This leads to melphalan resistance as well as perturbation of HR-related genes, including RAD51 [47]. Conversely, NER activity is more heterogenous in MM [48,49]. Slower NER activity and DNA DSB repair velocity correlate with increased melphalan effectiveness and accumulation of monoadducts [48]. Conversely, compound SCR7, via inhibition of enzymes involved in NER (including ERCC excision repair 3, TFIIH core complex helicase subunit-ERCC3/XPB or XPC complex subunit, DNA damage recognition and repair factor-XPC), sensitizes MM cells to melphalan treatment [48,49]. Reduction in the effectiveness of MMR leads to replication errors, which are especially common in DNA sequences with a repetitive structure (microsatellites or “short tandem repeats”). The proficiency of the MMR pathway seems to play a minor role in MM, with only a small subset of patients showing alteration of at least one microsatellite locus [50] or methylation of *MLH1* promoter [51,52].

Pathways involved in the repair of DNA DSBs are also abnormal in MM. Compared with RPMI-8226 parental cells, melphalan-resistant MM cell line LR5 cells have elevated expression of genes involved in the FA/BRCA pathway, such as BRCA1, BRCA2, FANCA, FANCC, FANCD2, FANCF, and RAD51, leading to reduced formation of ICLs and enhanced ICL removal [53]. LIG3 and PARP1 expression correlate with survival and are higher in certain high-risk MM subgroups [54,55,56]. PARP1 silencing or pharmacological inhibition using olaparib induces DNA damage and apoptosis, especially in *MYC*-high MM cells [55]. Moreover, LIG3 downregulation significantly reduces viability of MM cell lines, halting the LIG3-mediated alt-EJ pathway and increasing damaged DNA [54]. Both enforced expression of miR-22, a negative regulator of LIG3 [54], or LIG3 inhibition via the natural flavonoid Rhamnetin [57] impair DNA repair leading to MM cell death.

Anti-MM drugs also influence HR and NHEJ pathways either directly or indirectly. For instance, PD-L1 expression, which can promote HR repair [32], is increased by proteasome inhibitors and panobinostat [58,59] and decreased by immunomodulatory drugs [60,61]. Conversely, proteasome inhibitors can also induce a “BRCAness” state, with abrogation of H2A.X polyubiquitylation [56], and restrict FANCD2 and BRCA1 expression in cell lines and patients, potentially sensitizing them to melphalan and PARP inhibitors [56,62].

Finally, mRNA expression of WRN is also increased in MM patients compared with normal plasma cells. NSC19630, a specific WRN helicase inhibitor, induces increased DNA damage and apoptosis in preclinical models of MM [63].

## 3. Reactive Oxygen Species in DDR and Immunogenic Cell Death

Cancerous cells display an altered metabolism with an increase of ROS, which are short-lived molecules able to affect DDR [1,64]. At low concentrations, ROS can support growth and cell division; however, at high levels, ROS oxidize deoxyribonucleotide triphosphates (dNTPs), reducing replication fork velocity and causing fork replication breaks. This leads to DSBs, with ultimate onset of genomic instability in tumor cells. *ATM*-deficient cells have increased ROS [65] due to defects in NFE2 like bZIP transcription factor 2 (NFE2L2/NRF2) activity [66] and dysregulation of mechanistic target of rapamycin kinase (mTOR) as part of the mTORC1 complex [67]. Mitochondria produce the main sources of ROS (mROS) as a byproduct of electron transport chain activity [68]; however, other sites such as the endoplasmic reticulum (ER) can also release H_2_O_2_ during protein folding [69]. Yet, MM cells are able to survive under with very high levels of ROS, due to high mitochondrial oxidative metabolism, high metabolic rates by oncogenes such as *MYC* [39,70], and ER stress [71]. Therefore, MM cells need to tightly control their redox balance to avoid activation of various cell death pathways, such as apoptosis and autophagy triggered by excess ROS. PI induces a terminal unfolded protein response [72]; combining PIs with agents able to further induce oxidative stress leads to ROS-dependent apoptosis [73]. Bortezomib is also able to induce immunogenic cell death (ICD) [74], with ROS-based ER stress triggering pathways which govern the recruitment of cytotoxic T cells and dendritic cells to elicit anti-tumoral responses [75,76].

## 4. Mutations and Biomarkers of DNA Damage

### 4.1. Genomic Alterations Related to DDR and DNA Repair in MM

Loss-of-function mutations or deletion in *ATM* and in *ATR* have been reported in 2–4% of patients with sporadic MM [77,78], with cases of MM described in patients with ataxia telangiectasia. *TP53* variants (either del(17p) or *TP53* mutations) are present in 10–12% of cases at diagnosis and at higher rates at relapse, resulting in combined abnormalities in DDR in 15% of patients at diagnosis [78]. Patients with DDR abnormalities have reduced progression-free survival (PFS) and overall survival (OS), as described in the National Cancer Research Institute Myeloma XI trial [44]. Another specific mutational signature identified in MM which confers poor outcomes is the apolipoprotein B mRNA editing enzyme catalytic (APOBEC) signature [79,80]. The APOBEC3 family (A3A, A3B, A3C, and A3G) and activation-induced cytidine deaminase (AICDA/AID) are DNA-editing enzymes which act preferentially on single-strand DNA deaminating cytosines to uracil when cytosines immediately precede thymine (TpC context). This can lead to an enrichment of C > G and C > T mutations, a pattern associated with MAF bZIP transcription factor (*MAF*) translocations in MM [79,80]. Moreover, APOBEC3G via increasing HR activity is considered a driver for the acquisition of copy number variations and mutational changes in MM cells [81].

Polymorphism D693N within *BRCA1* and polymorphisms in *OGG1*, *ERCC1* (ERCC excision repair 1, endonuclease non-catalytic subunit), *ERCC4* (ERCC excision repair 4, endonuclease catalytic subunit), *XRCC1* (X-ray repair cross complementing 1), and *XRCC2* (X-ray repair cross-complementing 2) genes, all enzymes involved in DNA repair, are associated with responses to high-dose melphalan [82]. Moreover, polymorphisms in genes of the BER pathway increased the risk of developing MM (e.g., *OGG1* Ser326Cys) or conferred reduced OS (*APEX1* Asp148Glu and mutY DNA glycosylase-*MUTYH* Gln324) in a series of Japanese patients with MM [83]. Finally, first-degree relatives of Ashkenazi Jewish carriers of common *BRCA1* and *BRCA2* mutations tend to develop MM more commonly than the general population [84], and at least one family with multiple cases of MM has been linked to *BRCA2* mutations [85].

### 4.2. Biomarkers

High-dose melphalan is used as conditioning regimen for ASCT in myeloma. As with other alkylating agents, melphalan induces a range of cytotoxic and mutagenic adducts in DNA. Dimopoulos et al. confirmed the formation of monoadducts via melphalan therapy and reported that the area under the curve of total adducts in the peripheral blood mononuclear cells of patients post-melphalan is highly predictive of clinical responses [86]. However, melphalan also increases the general mutational burden in MM cells compared to bortezomib-lenalidomide-dexamethasone treatment [13,87]. Interestingly, patients achieving complete responses after ASCT have a significantly higher number of mutations than patients with less robust responses, possibly triggering neo-antigen formation and hence anti-tumoral immunity. However, the long-term repercussion of these mutations, especially in patients with previously unknown germline or acquired genetic abnormalities, is still vastly unexplored.

### 4.3. Relationship with Myeloid Neoplasm Conditions

Secondary primary malignancies (SPMs), especially acute leukemias or myelodysplastic syndrome, are not uncommon and occur with higher prevalence in patients with MM than in the general population [88]. This is partially due to host-related factors, genetic factors, and aging; however, an increase in the number of hematological SPMs has also been observed with co-exposure to lenalidomide and melphalan (especially with oral melphalan) [89]. Clonal hematopoiesis of indeterminate potential (CHIP), a condition characterized by the accumulation of leukemia-associated driver mutations in hematopoietic cells without underlying myeloid neoplasm [90], has also been reported in patients with MM at variable rates [91,92]. Interestingly, the presence of CHIP does not seem to impact MM outcomes or increase the risk of development of therapy-related myeloid neoplasms, possibly due to the lenalidomide used as maintenance post-ASCT. Therefore, the relationship between genomic instability, lenalidomide/melphalan use, and onset of secondary myeloid conditions is complex and warrant further prospective studies.

## 5. Therapeutic Interventions

Several compounds targeting either the DNA damage response or the DNA repair pathways are currently under investigation in clinical trials. Inhibitors of CHK1 and ATR have promising potential for cancer therapeutics, either alone or by sensitizing cells to DNA-damaging anti-MM agents [4]. The first natural substance to block ATR was found to be caffeine, even though its activity is weak. Different ATR inhibitors, such as M4344, Camonsertib, AZD6738 (Ceralasertib), and BAY1895344, are now in clinical trials for solid tumors, with the potential to also be tested in MM. In solid tumors, CHK1 inhibitors potentiate DNA-targeted chemotherapies, such as gemcitabine or irinotecan. AZD7762, an ATP-competitive CHK1/2 inhibitor, was shown to increase the action not only of alkylating agents such as melphalan and bendamustine, but also of bortezomib, especially in *TP53* mutated MM cells or those with 1q21 gains [93,94]. While AZD7762 development has been terminated, prexasertib (LY-2606368), another CHK1 kinase inhibitor, is undergoing active clinical trials in ovarian cancer, sarcomas, and solid tumors with replicative stress and homologous repair deficiencies, making it an interesting option in MM as well. Additionally, cancers with a defective G1 checkpoint are dependent on their G2 checkpoint, showing that WEE1 inhibition may sensitize cancer cells to anti-cancer agents [95], including CHK1 inhibitors [96]. Several WEE1 inhibitors (IMP7068, AZD1775, MK1775, or Debio 0123) are under development or in clinical trials for solid tumors and acute myeloid leukemia. MK1775 has also been tested in pre-clinical models impairing MM cellular growth and increasing the activity of bortezomib [97,98]; however, no clinical trials taking either approach have been created in MM. Finally, DNA-PK inhibitors, such as AZD7648 and peposertib, are also in clinical trials.

## 6. Final Considerations on DNA Repair Inhibitors and Their Clinical Use in MM

DDR is an important physiological process which becomes abnormal in several types of cancers, including MM. The presence of damaged DNA or defective DNA repair systems can have opposite effects on the survival of cancer cells. If the ability to mutate DNA leads to adaptation, evolution, and emergence of drug resistance or novel survival pathways, new antigens or neoepitopes can also be generated, potentially promoting immune surveillance. During MM development and progression, cancerous plasma cells acquire a collection of genetic mutations and abnormalities. The upregulation of DNA repair mechanisms to fix these DNA errors correlates with more aggressive disease and resistance to therapies. Similarly, the apoptotic DNA-damage response arm is often blunted by *TP53* or *TP73* abnormalities. Interestingly, some of the players involved in DNA repair, such as ATR, are less relevant in healthy normal tissues, but become a specific vulnerability of cancer cells in the setting of high DNA damage burden.

DNA repair inhibitors, such as ATR/CHK1/WEE1 inhibitors, can potentially be used in different settings in MM. Combination with proteasome inhibitors or melphalan will likely improve killing of MM cells, leading to deeper responses, while combination with immunotherapies might increase the production of new antigens and hence promote anti-tumoral immunity. Additionally, subsets of patients, such as those with *TP53* alterations or APOBEC signature, might greatly benefit from DNA repair inhibitors, which exploit a specific synthetic lethal vulnerability of these patients. On the other hand, DNA repair inhibitors can also be either too toxic or increase the risk of developing second malignancies, especially in individuals with personal or family history of other cancers or with concurrent CHIP. Therefore, DNA repair inhibitors might not be an ideal choice for patients with standard-risk disease who already have other therapeutic options. In conclusion, the field and understanding of DNA repair in MM and cancer in general is rapidly evolving. DNA repair inhibitors have potential as MM-directed therapies but need to be validated in science-driven clinical trials including specific subsets of patients with MM.
